# Cancer incidence among people living with HIV in Zimbabwe: A record linkage study

**DOI:** 10.1002/cnr2.1597

**Published:** 2021-12-07

**Authors:** Tinei Shamu, Eliane Rohner, Eric Chokunonga, Adrian Spoerri, Ardele Mandiriri, Cleophas Chimbetete, Matthias Egger, Julia Bohlius, Margaret Borok

**Affiliations:** ^1^ Newlands Clinic Newlands Harare Zimbabwe; ^2^ Institute of Social and Preventive Medicine University of Bern Bern Switzerland; ^3^ Graduate School of Health Sciences University of Bern Bern Switzerland; ^4^ Zimbabwe National Cancer Registry Parirenyatwa Group of Hospitals Harare Zimbabwe; ^5^ Centre for Infectious Disease Epidemiology and Research, School of Public Health and Family Medicine University of Cape Town Rondebosch Western Cape South Africa; ^6^ Population Health Sciences, Bristol Medical School University of Bristol Bristol UK; ^7^ Department of Epidemiology and Public Health Swiss Tropical and Public Health Institute Basel Switzerland; ^8^ University of Basel Basel Switzerland; ^9^ Unit of Internal Medicine, Faculty of Medicine and Health Sciences University of Zimbabwe Harare Zimbabwe

**Keywords:** cancer, HIV, incidence, record linkage, Zimbabwe

## Abstract

**Background:**

People living with HIV (PLWH) are at increased risk of developing cancer. Cancer diagnoses are often incompletely captured at antiretroviral therapy (ART) clinics.

**Aim:**

To estimate the incidence and explore risk factors of cancer in a cohort of PLWH in Harare using probabilistic record linkage (PRL).

**Methods:**

We conducted a retrospective cohort study that included PLWH aged ≥16 years starting ART between 2004 and 2017. We used PRL to match records from the Zimbabwe National Cancer Registry (ZNCR) with electronic medical records from an ART clinic in Harare to investigate the incidence of cancer among PLWH initiating ART. We matched records based on demographic data followed by manual clerical review. We followed PLWH up until first cancer diagnosis, death, loss to follow‐up, or 31 December 2017, whichever came first.

**Results:**

We included 3442 PLWH (64.9% female) with 19 346 person‐years (PY) of follow‐up. Median CD4 count at ART initiation was 169 cells/mm^3^ (interquartile range [IQR]: 82–275), median age was 36.6 years (IQR: 30.6–43.4). There were 66 incident cancer cases for an overall incidence rate of 341/100 000 PY (95% confidence interval [CI]: 268–434). Twenty‐two of these cases were recorded in the ZNCR only. The most common cancers were cervical cancer (*n* = 16; 123/100 000 PY; 95% CI: 75–201), Kaposi sarcoma, and lymphoma (both *n* = 12; 62/100 000 PY; 95% CI: 35–109). Cancer incidence increased with age and decreased with higher CD4 cell counts at ART initiation.

**Conclusion:**

PRL was key to correct for cancer under‐ascertainment in this cohort. The most common cancers were infection‐related types, reinforcing the role of early HIV treatment, human papillomavirus vaccination, and cervical cancer screening for cancer prevention in this setting.

**Abbreviations**
aHR: adjusted hazard ratio
AIDS: acquired immune deficiency syndrome
ART: antiretroviral therapy
CI: confidence interval
HIV: human immunodeficiency virus
HPV: human papillomavirus
HR: hazard ratio
IeDEA: International Epidemiology Database to Evaluate AIDS
IQR: interquartile range
MRCZ: Medical Research Council of Zimbabwe
PLWH: people living with HIV
PY: person‐years
RL: record linkage
USA: United States of America
WHO: World Health Organization
ZNCR: Zimbabwe National Cancer Registry

## 1 INTRODUCTION

People living with HIV (PLWH) are at increased risk of developing cancer.^1^, ^2^ Incidence rates of AIDS‐defining cancers such as Kaposi sarcoma and non‐Hodgkin lymphoma have been reduced by widespread access to antiretroviral therapy (ART), but late presentation for ART initiation is still a challenge despite current guidelines promoting a test‐and‐treat approach to ART initiation.^3^, ^4^, ^5^ PLWH who initiate ART at CD4 counts less than 350 cells/mm^3^ or with an AIDS defining condition are known to be at increased risk of developing cancer.^6^ Furthermore, HIV infection diminishes the ability to clear human papillomavirus (HPV) infection, which increases the risk of HPV related cancers, such as cervical cancer, among PLWH.^7^

The impact of HIV on cancer risk in sub‐Saharan Africa is still poorly understood.^8^ Reporting of cancer incidence among PLWH is often hampered by the absence of HIV serostatus information in national cancer databases, and cancer diagnoses are often incompletely captured at ART clinics.^9^ Record linkage methods have been widely used in high‐income countries but are not commonly used in low‐ to middle‐income countries due to low uptake of electronic health records systems by health institutions.^9^, ^10^, ^11^ Probabilistic record linkage (PRL), a method of linking records from different systems in the absence of a common unique identifier, have been used to improve under ascertainment of cancer incidence in sub‐Saharan Africa.^9^, ^10^, ^12^

In the current study, we estimated the incidence of cancer in a cohort of PLWH in Harare using PRL of records from the Zimbabwe National Cancer Registry (ZNCR) and an ART clinic, and explored risk factors for incident cancer in this population.

## 2 METHODS

We conducted a retrospective cohort study using datasets from the ZNCR and Newlands Clinic.

### 2.1 Study setting

The ZNCR is a population‐based registry for Harare. It is operated by the Ministry of Health and Child Care in Zimbabwe with the support of the International Agency for Research on Cancer. The ZNCR employs a combination of active and passive methods of case finding, with staff visiting institutions within the healthcare delivery system of Harare that are involved in the diagnosis and management of cancer patients to register cases. Death certificates of cancer patients who die in the greater Harare area are also used to identify cancer cases that may have been missed before death.^13^ A detailed description of the ZNCR operations is available elsewhere.^14^

The International epidemiology Databases to Evaluate AIDS Southern Africa (IeDEA) is an international research consortium of HIV observational databases, and the Southern African region (IeDEA‐SA, www.iedea-sa.org) includes ART programs located in seven countries (Botswana, Lesotho, Malawi, Zambia, Mozambique, Zimbabwe, and Republic of South Africa).^15^, ^16^ In this study, we used data from Newlands Clinic, one of the IeDEA‐SA sites situated in Harare, Zimbabwe. Newlands Clinic is a referral HIV management facility founded in 2004. It is a private voluntary organization that provides comprehensive HIV care to PLWH predominantly from the Greater Harare area, and is in a Public‐Private‐Partnership with the Ministry of Health and Child Care. It is operated by the Ruedi Luethy Foundation, a Swiss based charitable organization. Newlands Clinic's model of care comprises ART management, laboratory monitoring, mental and social health support, as well as sexual reproductive health (SRH) services. Under SRH services women who are sexually active are screened for cervical cancer. At the time of the study, cervical cancer screening was conducted using visual inspection with acetic acid (VIA). Women with a positive VIA screen were treated with cryotherapy or booked for a loop electrosurgical excision procedure (LEEP). Further details of Newlands Clinic's operations are available in a previous publication.^17^

### 2.2 Data collection and pre‐processing

We included all records available in the ZNCR and Newlands Clinic databases. The ZNCR database contained records up to December 2017. The ZNCR dataset stored in CanReg4 software (International Association of Cancer Registries, http://www.iacr.com.fr/) and Newlands Clinic's electronic medical records were exported and uploaded into KNIME Analytics Platform (Version 3.7.2, Build 18 April 2019). Pre‐processing of data was conducted using KNIME workflows built using core and custom KNIME nodes. Pre‐processing involved standardization of all strings to lower‐case, removal of special characters (other than alphabet characters and digits), stripping off white spaces and transformation of numerical values to strings. We used names, dates of birth, and national identification (ID) numbers, where available, for PRL.

### 2.3 Deduplication and record linkage

We used PRL to identify duplicate entries of the same patient in each of the two datasets. This involved linking each dataset to itself by comparing each record to other records in the same dataset using first names, middle names, last names, national identification numbers, year of birth, month of birth, and day of birth. There were no additional variables common to both databases that could be used for PRL. We compared names as complete texts as well as n‐grams. The variables used for record linkage were assessed for completeness. However, we added all records available into the deduplication and record linkage workflows regardless of their completeness. We used probability scores that were computed based on matching variables to classify records into matches, probable matches, and mismatches. We considered match pairs with a score of less than 12 as mismatches and those with scores >25 as definite matches.

We then linked deduplicated datasets from the ZNCR and Newlands Clinic to each other using the same variables. Records classified as matches or probable matches underwent another clerical review process. We used KNIME software for deduplication and PRL. Details of the record linkage parameters are shown in Appendix S1. Records were anonymized after PRL.

### 2.4 Statistical analysis and definitions

We used frequencies to describe patient characteristics and the spectrum of incident cancers diagnosed in PLWH starting ART between 2004 and 2017. We described continuous variables using medians and interquartile ranges (IQRs). We defined incident cancers as new cancer cases diagnosed more than 30 days after initiating ART. We categorized cancers that are typically associated with Group 1 infectious agents (classified as carcinogenic to humans by the International Agency for Research on Cancer) as infection related.^18^

For the analysis of cancer incidence, we included all patients aged ≥16 years, who started ART at Newlands Clinic between 2004 and 2017. We excluded patients diagnosed with cancer before ART initiation or within 30 days after (considered as prevalent cancer cases), and all patients with follow‐up time of <30 days from ART initiation. We calculated person‐years (PY) at risk from 30 days after ART initiation until the date of first cancer diagnosis, death, 6 months after the last visit for patients who did not die, or 31 December 2017, whichever occurred first. We present crude cancer incidence rates per 100 000 PY with 95% confidence intervals (CI). The confidence intervals were calculated by quadratic approximation to the Poisson log likelihood for the log‐rate parameter.^19^

We used univariable and multivariable Cox proportional hazards models to assess the following potential risk factors (exposure variables) for developing cancer: sex, age at ART initiation (continuous variable), ART initiation period, education, employment status, CD4 count category at ART initiation (<100, 100–199, ≥200 cells/mm^3^) and WHO clinical stage at ART initiation. Demographic characteristics were as collected at patient registration into the Newlands Clinic cohort. The outcome variable (cancer diagnosis) was defined as having a cancer diagnosis recorded in either the ZNCR or Newlands Clinic database. In the multivariable model, we included sex, education and CD4 count at the time of ART initiation. We did not include WHO clinical stage at ART initiation in the multivariable model due to collinearity with CD4 cell count. We tested proportional hazard assumptions individually for each covariate using Schoenfeld residuals. All statistical analyses were conducted using Stata version 13.1 (StataCorp, College Station, Texas 77 845 USA).

## 3 RESULTS

**FIGURE 1:**
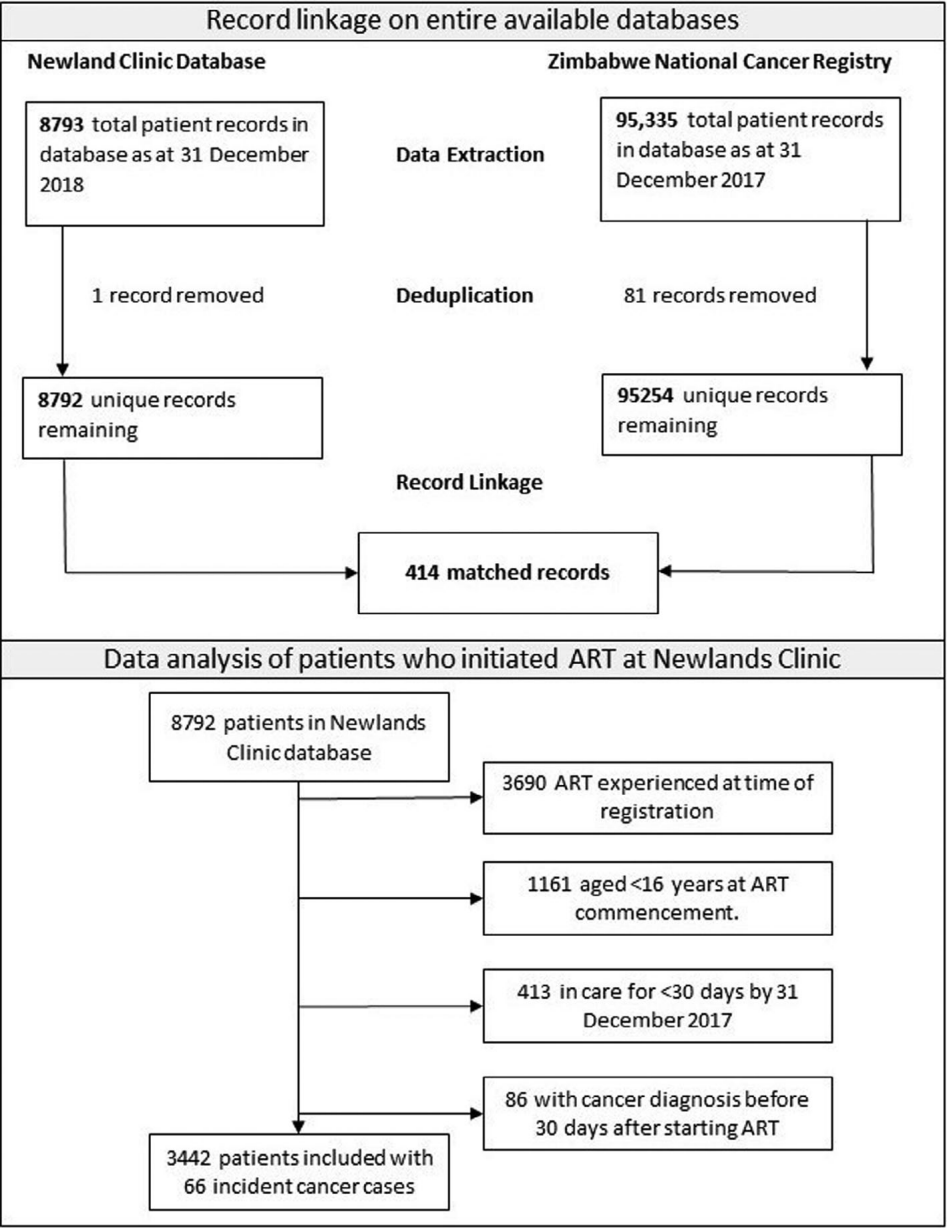
Top: Flow chart of record linkage between Zimbabwe National Cancer Registry and Newlands Clinic; Bottom: Flow diagram of patients included in the study

After deduplication, 8792 records from the Newlands Clinic and 95 254 records from the ZNCR were probabilistically linked resulting in 414 confirmed matches (Figure 1). Details of the PRL dataset completeness are given in Appendix S2. Within this linked dataset, 5102 PLWH had newly initiated ART at Newlands Clinic between 2004 and 2017. We excluded 1161 children younger than 16 years, 413 individuals with less than 30 days of follow‐up after ART initiation, and 86 patients with a prevalent cancer diagnosis (Figure 1).

For the cancer incidence analysis, we included 3442 PLWH with 66 incident cancers. Thirty cancer cases (45%) were recorded in both databases, whereas 22 cases (33%) were documented only in the ZNCR database, and 14 cases (21%) were found only in the Newlands Clinic database (Appendix S3). Of the 14 cases found only in the Newlands Clinic database, 10 had documented biopsy information while visit notes were not available for the other four. Two‐thirds of the included PLWH were female (2235, 64.9%) and the median age at ART initiation was 36.6 years (IQR 30.6–43.4). About a 10th were aged over 50 years (374, 10.9%) and most had secondary or tertiary education (2609, 75.8%), although only 1261 (36.6%) were employed. Most of the included PLWH were severely immune compromised with a median CD4 cell count of 169 cells/mm^3^ (IQR 82–275) at ART initiation. Almost a third (1039, 30.2%) had CD4 cell counts less than 100 cells/mm^3^. Forty‐one percent (*n* = 1404) were classified as WHO clinical stage three or four at ART initiation (Table 1).

**TABLE 1:** Characteristics and cancer incidence rates of patients initiating antiretroviral therapy at Newlands Clinic between 2004 and 2017 (*N* = 3442)

Characteristic	Patients *n* (%)	Person‐years at risk	Cancer cases (*n*)	Incidence rate per 100 000 PY (95% CI)
All	3442	19 346	66	341 (268–434)
Sex				
Female	2235 (64.9)	12 994	50	385 (292–508)
Male	1207 (35.1)	6351	16	252 (154–411)
Age group (years)				
16–35	1489 (43.3)	8275	22	265 (175–404)
36–50	1579 (45.9)	9053	34	376 (268–526)
> 50	374 (10.9)	2018	10	496 (266–921)
ART initiation period (years)				
2004–2007	653 (19.0)	6344	20	315 (203–489)
2008–2010	944 (27.4)	7025	26	370 (252–544)
2011–2013	761 (22.1)	3834	13	339 (197–584)
2014–2017	1084 (31.5)	2143	7	327 (156–685)
Education				
Primary or none	833 (24.2)	5478	26	475 (323–697)
Secondary or tertiary	2609 (75.8)	13 868	40	288 (212–393)
Employment				
Employed	1261 (36.6)	6564	23	350 (233–527)
Unemployed	1970 (57.2)	11 396	40	351 (257–479)
Unknown	211 (6.1)	1386	3	‐
CD4 count categories at ART initiation (cells/mm^3^)				
<100	1039 (30.2)	6271	33	526 (374–740)
100–199	974 (28.3)	6397	17	266 (165–427)
≥200	1410 (41.0)	6656	16	240 (147–392)
Unknown	19 (0.6)	21	0	‐
WHO clinical stage for HIV disease at ART initiation				
Stage 1	1144 (33.2)	4915	10	203 (109–378)
Stage 2	890 (25.9)	5488	17	310 (193–498)
Stage 3	1061 (30.8)	6952	29	417 (290–600)
Stage 4	343 (10.0)	1977	10	506 (272–940)
Unknown	4 (0.1)	14	0	‐

*Note*: Incidence rates are unadjusted. Data source: Linked Zimbabwe National Cancer Registry and Newlands Clinic electronic records.

Abbreviations: ART, antiretroviral therapy; CI, confidence interval; PY, person‐years.

Patients who developed cancer were slightly older at ART initiation (median age 39.1 years, IQR 33.8–45.5) compared with those who did not develop cancer (median age 36.5 years, IQR 30.4–43.3). The median baseline CD4 count among patients who developed cancer was lower (106 cells/mm^3^, IQR 54–196) than among those who did not (170 cells/mm^3^, IQR 84–276), *p* = .001. Half of the PLWH who developed an incident cancer (*n* = 33, 50.0%) had initiated ART with CD4 counts below 100 cells/mm^3^.

Overall, there were 66 incident cancer cases during 19 346 PY of follow‐up for an incidence rate of 341/100 000 PY (95% CI 268–434). The cancer incidence rate increased with more advanced WHO clinical stage and decreasing CD4 counts at ART initiation (Table 2). PLWH who initiated ART at a CD4 count of <100 cells/mm^3^ had a particularly high cancer incidence rate of 526/100 000 PY (95% CI 374–740). The most common cancer was cervical cancer (*n* = 16) with an incidence rate of 123/100 000 female PY (95% CI 75–201) followed by Kaposi sarcoma and lymphoma with 12 cases each (62/100 000 PY; 95% CI 35–109). The distribution of the remaining cancer cases was as follows: five non‐cervical anogenital cancer (26/100 000 PY; 95% CI 11–62), five breast, three conjunctiva, two central nervous system, five gastrointestinal, and one each of kidney, larynx, lung, plasma cell tumor, soft tissue sarcoma, and uterus.

**TABLE 2:** Risk factors for cancer diagnosis after antiretroviral therapy initiation

Characteristic	HR (95% CI)	aHR (95% CI)	*p*‐Value
Sex			
Male	1	1	0.053
Female	1.56 (0.89–2.74)	1.76 (0.99–3.12)	
Age at ART initiation	1.03 (1.01–1.05)	**1.03 (1.00–1.05)**	**0.024**
ART initiation period			
2004–2007	1	‐	
2008–2010	1.14 (0.61–2.12)	‐	
2011–2013	0.90 (0.43–1.90)	‐	
2014–2017	0.61 (0.24–1.54)	‐	
Education			
Primary or none	1	1	0.231
Secondary or tertiary	**0.57 (0.35–0.94)**	0.73 (0.44–1.22)	
Employment			
Unemployed	1	‐	
Employed	0.97 (0.58–1.62)	‐	
CD4 count categories at ART initiation (cells/mm^3^)			
<100	1	1	**0.007** **^a^**
100–199	**0.51 (0.28–0.92)**	**0.49 (0.27–0.88)**	
≥200	**0.42 (0.23–0.77)**	**0.42 (0.23–0.77)**	
WHO clinical stage at ART initiation			
Stage 1	1	‐	
Stage 2	1.70 (0.78–3.73)	‐	
Stage 3	**2.33 (1.13–4.82)**	‐	
Stage 4	**2.77 (1.15–6.68)**	‐	

*Note*: Covariates without statistically significant results in the univariable model were not included in the multivariable model. WHO stage was not included in the multivariable model due to collinearity with CD4 cell count. Bold type indicates statistically significant results. *p*‐Values are from the multivariable analysis model.

Abbreviations: aHR, adjusted hazard ratio; ART, antiretroviral therapy; HR, hazard ratio.

a From likelihood ratio test.

In adjusted analyses, the risk of developing cancer increased by 3% with each additional year in age at ART initiation (adjusted hazard ratio [aHR] 1.03, 95% CI 1.00–1.05). When compared with PLWH commencing ART with CD4 cell counts below 100 cells/mm^3^, those with CD4 cell counts between 100 and 199 had a 51% lower risk (aHR 0.49, 95% CI 0.27–0.88) while those with CD4 cell counts ≥200 cells/mm^3^ had a 58% lower risk of developing cancer (aHR 0.42, 95% CI 0.23–0.77), see Table 2. Cancer risk tended to be higher in female than male PLWH (aHR 1.76, 95% CI 0.99–3.12).

## 4 DISCUSSION

We report record linkage ascertained cancer incidence rates from an HIV cohort in Zimbabwe showing that cervical cancer was the most common incident malignancy with an incidence rate of 123 per 100 000 PY. Most incident cancer cases were AIDS‐defining malignancies. The risk of being diagnosed with cancer increased with older age and lower CD4 cell count at ART initiation.

The strength of our study was the use of PRL methods to enhance cancer ascertainment at an ART clinic. We matched quality controlled electronic medical records at Newlands Clinic to cancer records from the ZNCR and, thereby, managed to identify an additional 22 cancer cases that were only recorded in the ZNCR. This enabled us to conduct what to our knowledge is the first record linkage ascertained cancer incidence study in Zimbabwe. However, there were some limitations to the study. The Newlands Clinic cohort is relatively small compared with those that have been analyzed in similar studies^9^, ^10^ and not necessarily representative of the much larger public ART clinics in Zimbabwe. Furthermore, many PLWH who were referred to Newlands Clinic had initiated ART elsewhere and were, therefore, excluded from this analysis, further reducing the sample size. In addition, our results may be an underestimate in view of the high number of records with incomplete key data such as missing national ID numbers, full names, or dates of birth (Appendix 2).

In line with similar studies from Malawi and South Africa,^9^, ^10^ we found AIDS‐defining cancers to be the most common cancer diagnoses among PLWH. However, our overall cancer incidence rate estimate was lower than that observed in Malawi (age‐adjusted incidence: 689 per 100 000 PY, 95% CI: 610–768) and South Africa (age‐standardized incidence: 877 per 100 000 PY, 95% CI: 744–1041). Of note, the three studies may not be directly comparable due differences in study periods, study populations, and definition of incident cancers. Furthermore, we reported crude incidence rates, which may be lower than age‐adjusted rates given the young age of our cohort. Interestingly, Kaposi sarcoma was by far the most common cancer diagnosis in the other two studies,^9^, ^10^ but incidence rates in our study were highest for cervical cancer. This is in line with a report that showed four‐fold cumulative incidence of cervical cancer compared with Kaposi sarcoma in the general population of Harare and Bulawayo, Zimbabwe, between 2011 and 2013.^20^ Active screening of women for cervical cancer since 2011 may also have contributed to increased case finding at Newlands Clinic.

Infection related cancers such as cervical and other anogenital cancers, Kaposi sarcoma, and lymphoma accounted for most incident cancers in our study. Infectious agents are common causes of cancer in sub‐Saharan Africa, unlike in high‐income settings where lifestyle factors are the predominant causes of cancer.^3^, ^8^ The high burden of HPV associated cancers in our study reinforces the urgency of accelerating HPV vaccination programs to advance toward the 90‐70‐90 targets of eliminating cervical cancer as a public health problem by 2030.^21^, ^22^ It also highlights the important role of cervical cancer screening programs to reduce the incidence of cervical cancer.^23^, ^24^ Furthermore, consistent with other studies, our results showed that initiating ART at higher CD4 cell counts reduced the overall risk of developing cancer.^25^, ^26^ Enhanced efforts toward HIV testing and early ART initiation as prescribed in the WHO “test‐and‐treat” drive is likely to reduce cancer incidence among PLWH.

Linking records between the ZNCR and Newlands Clinic improved cancer incidence ascertainment by identifying cancer cases that were recorded in only one of either of the databases. However, of 14 patients whose cancer diagnoses were only found in the Newlands Clinic database, 10 patients had histological information available. For these patients, the failure to find matching records in the ZNCR was probably due to missing or incomplete information that impeded the PRL process. Active investment in electronic medical records systems is needed to improve gathering of epidemiologic data in low‐ and middle‐income countries. Reliable epidemiologic data are essential to inform allocation of resources and planning of interventions. However, as observed in our study, complete and accurate capturing of identifying variables such as names and national ID numbers is needed to achieve accurate record linkage results. This effort involves all stakeholders in the chain of care including ART and oncology clinics, laboratories, radiology service providers and cancer registry data capture clerks.

In conclusion, we used PRL methods to improve the estimation of cancer incidence among PLWH in Zimbabwe. Cervical cancer and other infection related cancers were the most common cancer types in this cohort of PLWH on ART. Lower CD4 cell counts at the time of ART initiation and older age were associated with higher risk of developing cancer. Intensified efforts toward HPV vaccinations and the WHO prescribed “test‐and‐treat” approach for early ART initiation may help lower cancer incidence among PLWH.

## CONFLICT OF INTEREST

The authors declare no conflict of interest.

## AUTHOR CONTRIBUTIONS

*Conceptualization, Formal Analysis, Methodology, Project Administration, Supervision, Visualization*, E.R.; *Conceptualization, Data Curation, Methodology, Resources*, E.C.; *Data Curation, Methodology*, A.M.; *Conceptualization, Data Curation, Formal Analysis, Methodology, Software*, A.S.; *Conceptualization, Methodology, Project Administration, Supervision*, C.C.; *Conceptualization, Funding Acquisition, Methodology, Resources, Supervision*, M.E.; *Conceptualization, Funding Acquisition, Methodology, Supervision*, J.B.; *Conceptualization, Investigation, Methodology, Resources, Supervision*, M.B.

## ETHICAL STATEMENT

Participants included in this study provided written consent. The study was approved by the Newlands Clinic Research Team and the Medical Research Council of Zimbabwe (MRCZ No. A1336).

## Supporting information


**Appendix S1.** Supporting Information.

**Appendix S2.** Supporting Information.

**Appendix S3.** Supporting Information.

## Data Availability

The data that support the findings of this study are available from the corresponding author upon reasonable request.
