# Supplementary material for: Cancer incidence among people living with HIV in Zimbabwe: A record linkage study
Source: Cancer Rep (Hoboken). 2021 Dec 7;5(10):e1597. doi: 10.1002/cnr2.1597 (PMC9575496; doi:10.1002/cnr2.1597)
Supplement: Supplementary file 1 — Appendix S1. Supporting Information. [file CNR2-5-e1597-s001.docx]

**Appendix S1**

**Probabilistic record linkage**

| Software | Konstanz Information Miner KNIME (<https://www.knime.com>), with our own K-Link scripts and nodes for Bloom filtering and PRL. |
| --- | --- |
| Method | The linkage is based on the Fellegi-Sunter model^[[1]](#footnote-1)^ and Bloom filters for similarity measures and blocking^[[2]](#footnote-2)^. |
| Blocking | The blocking method implemented was based on a Bloom filter including first name, last name, and date of birth. Record pairs with a Tanimoto coefficient >0.4 were considered as potential linked pairs and used for the following linkage procedures. |
| Linkage rules | Rules included agreement, disagreement and missing as outcome. For first name, last name, and the national ID we additionally used a Bloom filter and a Tanimoto coefficient to allow for typos. |
| Outcome weights | The software calculated the u-probability based on a sample of 250'000 randomly matched pairs. The m-probability was estimated based on the set of potential matched pairs in 3 iterations of applying rules, calculating weights, and deleting pairs with a low total linkage weight.  The total linkage weight was calculated as sum of log_2_(m-probability) + log_2_(u-probability). |
| Cut-offs, matches and clerical review | From the set of potential matches we excluded pairs with a total weight <12. Records with total weight >24 were considered as definite matches in the deduplication process and those in between were checked by clerical review. In the final record linkage, all records with total weight ≥12 were checked by clerical review and classified individually as match or non-match. |

1. Fellegi P, I., & Sunter B, A. (1969). A theory of record linkage. *Journal of the American Statistical Association*, *64*(328), 1183–1210. [↑](#footnote-ref-1)
2. Bachteler, T., Reiher, J., & Schnell, R. (2013). Similarity Filtering with Multibit Trees for Record Linkage. In *German Record Linkage Center, working papers series* (p. 20). [↑](#footnote-ref-2)
