# Supplementary material for: Cancer incidence among people living with HIV in Zimbabwe: A record linkage study
Source: Cancer Rep (Hoboken). 2021 Dec 7;5(10):e1597. doi: 10.1002/cnr2.1597 (PMC9575496; doi:10.1002/cnr2.1597)
Supplement: Supplementary file 2 — Appendix S2. Supporting Information. [file CNR2-5-e1597-s002.docx]

**Appendix S2**

**Dataset Characteristics**

| **Variable** | **Zimbabwe National Cancer Registry** | **Newlands Clinic** |
| --- | --- | --- |
|  | **N (%)** | **N (%)** |
| Total Records | 95,254 (100) | 8,792 (100) |
| First Names^¥^  Total Available  Initials only  Missing | 94,081 (100)  1,169 (1.2)  4 (0.0) | 8,792 (100)  0  0 |
| Last Names  Total Available  Missing | 95,252 (100)  2 (0.0) | 8,792 (100)  0 |
| National Identification Number^£^  Total Available  Complete  Incomplete (Partial)  Missing | 40,613 (42.6)  36,833 (38.7)  3,780 (4.0)  54,641 (57.4) | 5,862 (66.7)  5,067 (57.6)  795 (9.0)  2,930 (33.3) |
| Year of birth  Total Available  Missing | 68,597 (72.0)  26,657 (28.0) | 8,792 (100)  0 |
| Month of birth  Total Available  Missing | 68,556 (72.0)  26,698 (28.0) | 8,792 (100)  0 |
| Day of birth  Total Available  Missing | 68,554 (72.0)  26,700 (28.0) | 8,792 (100)  0 |

^¥^First names consisting of a single letter were classified as initials only.

^£^Zimbabwean National Identification numbers were expected to have 10 numeric characters and one alphabetic character on the ninth position after removing spaces. Identity numbers with less than characters as well as those missing an alphabetic character were classified as incomplete.
