# Supplementary material for: Cancer incidence among people living with HIV in Zimbabwe: A record linkage study
Source: Cancer Rep (Hoboken). 2021 Dec 7;5(10):e1597. doi: 10.1002/cnr2.1597 (PMC9575496; doi:10.1002/cnr2.1597)
Supplement: Supplementary file 3 — Appendix S3. Supporting Information. [file CNR2-5-e1597-s003.docx]

**Appendix S3**

List of cancer diagnoses in the Zimbabwe National Cancer Registry, Newlands Clinic, and both (N = 66)

| **Diagnosis** | **NC Only** | **ZNCR Only** | **Both** | **Total** |
| --- | --- | --- | --- | --- |
| Cervical cancer | 2 | 2 | 12 | 16 |
| Kaposi sarcoma | 4 | 5 | 3 | 12 |
| Lymphoma | 4 | 5 | 3 | 12 |
| Anogenital cancer (non-cervical) | 1 | 1 | 3 | 5 |
| Breast cancer | 2 |  | 3 | 5 |
| Gastrointestinal cancer | 1 |  | 3 | 4 |
| Conjunctival cancer |  | 2 | 1 | 3 |
| CNS tumor |  | 2 |  | 2 |
| Esophageal cancer |  | 1 |  | 1 |
| Kidney cancer |  | 1 |  | 1 |
| Laryngeal cancer |  | 1 |  | 1 |
| Lung cancer |  | 1 |  | 1 |
| Uterine cancer |  | 1 |  | 1 |
| Plasma cell tumor |  |  | 1 | 1 |
| Soft tissue sarcoma |  |  | 1 | 1 |
| **Total, n (%)** | **14 (21.2)** | **22 (33.3)** | **30 (45.5)** | **66 (100)** |

NC: Newlands Clinic, ZNCR: Zimbabwe National Cancer Registry, CNS: Central nervous system
